# The relationship between parental support for exercise and depression: The mediating effects of physical exercise and physical self-esteem

**DOI:** 10.1371/journal.pone.0304977

**Published:** 2024-06-25

**Authors:** Chao Wang, Yonghua Luo, Hansen Li, Guodong Zhang

**Affiliations:** 1 College of Physical Education and Sports, Southwest University, Chongqing, China; 2 Youth League Committee of Hotan Normal College, HeTian Normal College, Hetian, China; 3 Second Middle School, Suining City, Sichuan, China; Dilla University College of Health Sciences, ETHIOPIA

## Abstract

The mental health challenges among Chinese college students have become a pressing social concern. This study examined the relationship between parental support for exercise and depression among freshmen and also explored the mediating role of physical exercise and physical self-esteem. Utilizing the Parental Exercise Support Scale, Depression Self-Rating Scale, Physical Activity Rating Scale, and Physical Self-Esteem Scale, a questionnaire survey was conducted. Convenient samples from two universities were recruited by university teachers, which included 766 university freshmen. Correlation and linear regression analyses were employed to assess the overall associations while bootstrapping method was used to test mediation effects. Results indicated significant correlations between parental support for exercise and physical exercise, physical self-esteem, and depression. Physical exercise and physical self-esteem were found to mediate the relationship between parental support for exercise and depression, both individually and sequentially. These findings highlight the potential association between parental support for exercise and the mental health of college freshmen and also offer a mechanism to understand this association.

## Introduction

With the increase in university admissions, college students have become a large group. As of 2023, the number of college students in China has exceeded 30 million. According to the "China’s National Mental Health Report 2022" by the Chinese Academy of Sciences, around 21.48 percent of students showed signs of depression, and about 45.28 percent showed signs of anxiety [1]. This highlights the importance of addressing the mental health of college students. College freshmen, in particular, are vulnerable to mental health issues. Research indicates that first-year students often experience depression during their adjustment to university life [2]. This may be resulted from the changes in identity and the challenges they encounter during this period. Given these factors, it is imperative to explore depression among freshmen to support their mental well-being.

Currently, many studies are exploring factors that may influence the mental health of college students in order to develop strategies for promoting mental health. Known factors include region, biology, income, personality, and personal experiences, among others [3–5]. Among these, the family is an important factor [6–9]. For example, parental involvement behaviours, such as connection, bonding, engagement, and support, may promote mental health and reduce the risk of depression [10]. Moreover, some studies have suggested that higher levels of parental involvement (e.g., parental care and communication) in childhood are associated with a lower risk of depression in later life [11–13]. Parental involvement also has cumulative benefits in preventing depression. However, parental support for exercise in particular is lacking in research. Is parental support for exercise related to the mental health of college students? Through what pathways? These questions remain to be answered. In response, we investigate the potential impact of parental support in promoting exercise on depression among freshmen.

### Parental support for exercise, physical exercise, and depression

Parental guidance and support typically begin from infancy and remain crucial throughout individuals’ lives [14]. It’s widely recognized that parental support plays a pivotal role in shaping mental well-being [15, 16]. The negative association between parental support and depression has also been widely documented [17–19]. Parental support for exercise, as a distinct form of support, offers unique mental health benefits. On one hand, physical exercise can become a habit that persists throughout individuals’ lifespans [20]. Therefore, parental support for their children’s physical exercise may not only influence their current level of physical activity but also their subsequent physical exercise in adulthood [21]. On the other hand, physical exercise has shown great potential in mitigating negative moods and mental health issues [22, 23]. Therefore, increased levels of physical exercise due to parental support may eventually lead to lower levels of mental health issues.

### Physical exercise, physical self-esteem, and depression

Meanwhile, a close relationship exists between physical exercise and body self-esteem. As early as the 1980s, psychologists developed a model illustrating the interaction between physical activity and body self-esteem. The model elucidates the pathways through which physical activity affects physical self-esteem, highlighting four key areas: motor skills, physical condition, physical fitness, and physical attractiveness [24]. Furthermore, prior studies have indicated a connection between physical self-esteem and depression [25–27]. In additional mediation analyses, self-esteem was discovered to mediate the link between physical activity or sports and depressive symptoms.

**Fig 1:**
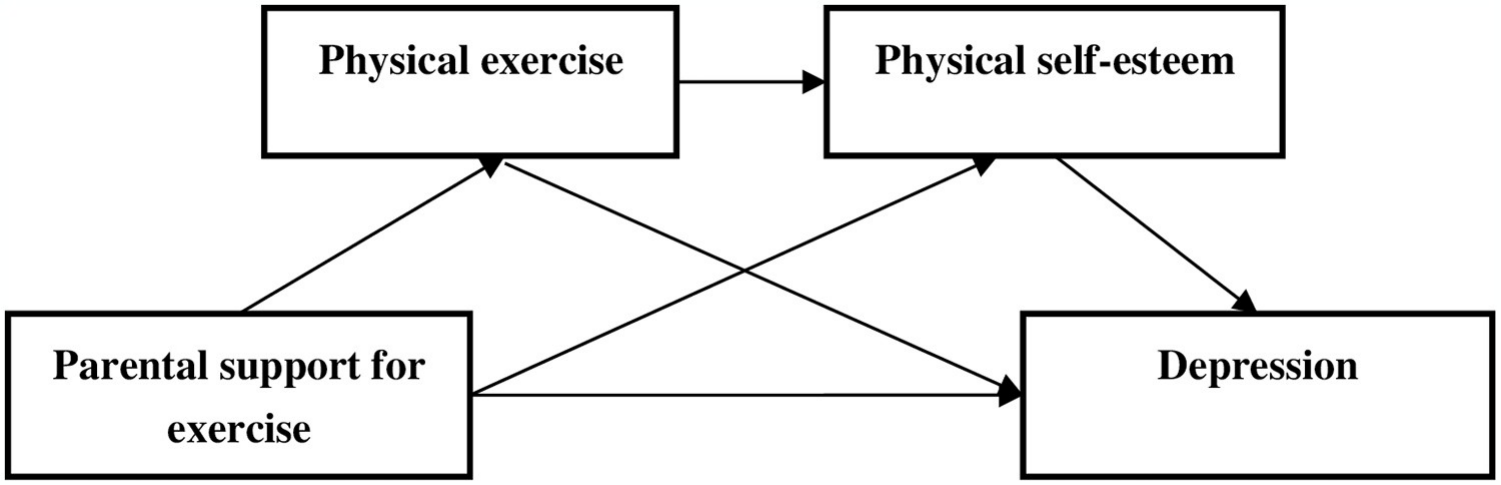
Chain intermediary model schematic.

Based on the aforementioned studies, we aim to establish a model that elucidates the relationship between parental support for exercise and depression. The conceptual framework of our study is outlined below (Fig 1):

## Materials and methods

### Participants and procedure

Our target participants are university freshmen. Therefore, our inclusion criteria were: (1) First-year freshmen enrolled in 2021; and (2) Raised by both parents or one parent. Exclusion criteria were: (1) Non-sports majors (to exclude the influence of specialized training) and (2) Individuals with severe health problems that forbid physical activity or exercise. We invited teachers from two universities to promote the survey among freshmen and recruit participants. Recruited students filled out the survey online during breaks between classes. A total of 780 individuals participated in the survey, and after removing ineligible questionnaires, 766 individuals were included in the analysis. Participation in this study was purely voluntary. We introduced our plan and distributed recruitment messages to the detained people through routine meetings at the correctional center. Written informed consent to participate in this study was provided by our participants or their parents (for participants aged below 18).

### Research instrument

The survey questionnaire used in this study consists of four scales. They are the Parental Exercise Support Scale, the Self-Rating Depression Scale (SDS), the Physical Activity Level Scale, and the Physical Self-Perception Profile (PSPP).

The Parental Exercise Support Scale [28] was developed by Gesier, referring to the classification of parental support by Beets and other scholars, as well as previous viewpoints. This scale consists of four dimensions with a total of 13 items. The scale adopts a Likert 7-point scoring method, ranging from "never" to "always" based on self-perception. A higher total score indicates a higher level of parental support. In this study, the α coefficient of this scale is >0.7.

The Self-Rating Depression Scale (SDS) [29] was developed by Zung and consists of 20 items primarily used to measure an individual’s depressive state. It adopts a 4-point scoring method, with scores from the 20 items summed to obtain a total score. A higher total score indicates a higher level of depression. In this study, the α coefficient of this scale is >0.7.

The Physical Activity Level Scale is a revised version based on the work of Professor Liang Deqing [30] and others from Wuhan Institute of Physical Education, building upon the original version developed by Hashimoto Kimokuma in Japan. This scale consists of three items, with answers for each item divided into five levels and scores ranging from 1 to 5, measuring the intensity, duration, and frequency of exercise. In this study, the α coefficient of this scale is 0.7.

The Physical Self-Perception Profile (PSPP) was revised by Xu Xia [31], specifically designed for university freshmen, and includes a main scale—Physical Self-Worth—and four subscales—Physical Skill, Physical Condition, Physical Attractiveness, and Physical Fitness. This scale is specifically suitable for the Chinese population. In this study, the α coefficient of this scale is >0.7.

### Statistical analysis

We used Harman’s one-way test to check for the potential common method bias in the questionnaire. The core factors we extracted explained 35.64% of the total variance, which is below the recommended reference value of 40%. Additionally, we constructed a single-factor confirmatory factor analysis model, which did not demonstrate acceptable fit indices (χ^2^/df = 50.780, RMSEA = 0.255, CFI = 0.674, NFI = 0.670, and TFL = 0.615). These suggested a relatively low risk of common method bias in this study.

We assessed the correlations between several variables in the mediation model using Pearson’s linear correlation coefficients.

To validate the mediation effects, we used the bias-corrected percentile Bootstrap method with 5000 repetitions to generate 95% confidence intervals. All analyses were conducted using SPSS 25, AMOS 25, and PROCESS 4.0. According to MacCallum et al. [32], the sample size for structural equation modeling (e.g., chain mediation model) should be ten times the number of model parameters or more. Based on the parameters (n = 10) required to estimate in the framework, we should have at least 100 samples. Therefore, our sample size of 766 was sufficient.

In this study, two-tailed p-values less than 0.05 were considered statistically significant evidence.

## Results

### Participants’ characteristics

Our sample consisted of 766 individuals, with a slightly higher proportion of females (Table 1). The average age was around 19 years old, and the average height was 168 cm. There were 446 of them from Southwest University and 320 from Northwestern University.

**Table 1:** Demographic characteristics descriptive statistics.

Quorum	Male	Female	Age(year)	Height(cm)
***766***	314	452	19.1±1.37	168.46±8.22

### Correlation analysis between variables

As shown in Table 2, parental support for exercise was positively correlated with physical exercise and physical self-esteem (r = 0.399, p<0.01; r = 0.297, *p*<0.01) and negatively correlated with depression (r = -0.296, *p*<0.01). Physical exercise was significantly positively correlated with physical self-esteem (r = 0.628, *p*<50.01) and negatively correlated with depression (r = -0.531, *p*<0.01). Physical self-esteem was significantly negatively associated with depression (r = -0.446, *p*<0.01).

**Table 2:** Correlation analysis between variables.

Variables	M	SD	1	2	3	4
***Parental exercise support***	54.730	18.720	-			
***Physical exercise***	14.792	19.242	0.399**	-		
***Physical self-esteem***	66.650	23.553	0.297**	0.628**	-	
***Depression***	56.909	15.019	−0.296**	−0.531**	−0.446**	-

Note:

* p<0.05,

** p<0.01

### Mediation analysis

**Fig 2:**
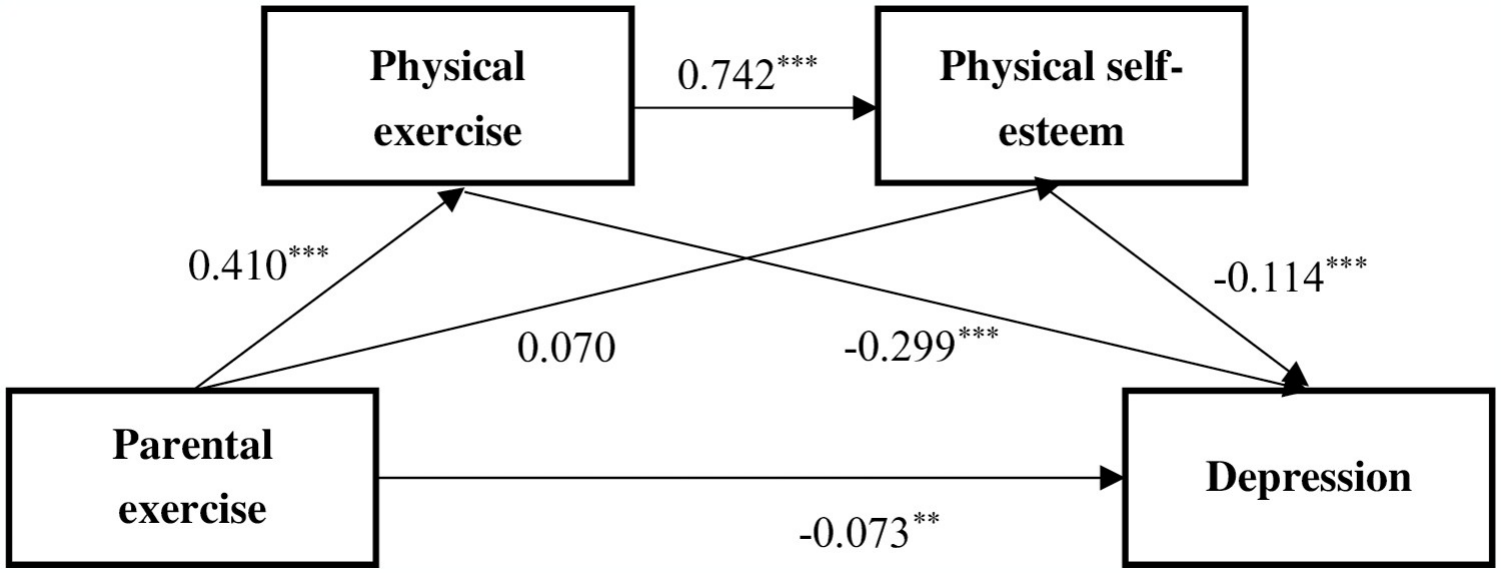
Intermediary effect model diagram.

Table 3 and Fig 2 show the total, direct, and indirect effects of parental exercise support on depression. The total, direct, and indirect effects of parental exercise support on depression were statistically significant (indicated by the 95% confidence interval), and the indirect effect accounted for 69.6% of the total effect. Specifically, the “Parental support for exercise->Physical exercise->Depression” chain was the main contributing pathway, followed by the pathway “Ind3”, which was mediated by both physical exercise and body self-esteem.

**Table 3:** Analysis of mediating effects of physical exercise and physical self-esteem.

Type of effect	Efficacy value	Boot SE	Boot LLCI	Boot ULCI
***Total effect***	−0.237	0.028	−0.292	−0.183
***Direct effect***	−0.073	0.026	−0.124	−0.021
***Total indirect effect***	−0.165	0.019	−0.203	−0.129
***Ind1***	−0.122	0.018	−0.158	−0.090
***Ind2***	−0.008	0.005	−0.018	0.001
***Ind3***	−0.035	0.007	−0.049	−0.020
***(C1)*: *Ind1-Ind2***	−0.114	0.018	−0.152	−0.079
***(C2)*: *Ind1-Ind3***	−0.088	0.020	−0.128	−0.052
***(C3)*: *Ind2-Ind3***	0.027	0.008	0.012	0.045

Note: Ind1: Parental support for exercise->Physical exercise->Depression

Ind2: Parental support for exercise t->Body self-esteem->Depression

Ind3: Parental support for exercise -> physical exercise -> body self-esteem -> depression

## Discussion

The purpose of this study is to explore the relationship between parental support for physical exercise among college freshmen and depression, as well as the mediating role of physical exercise and physical self-esteem. As hypothesized in our theoretical model, parental support for physical exercise was negatively correlated with depression. Secondly, physical exercise and physical self-esteem formed a chain mediation pathway, partially explaining the overall association. This finding underscores the importance of parental support for their children’s exercise.

### Parental exercise support and depression

There is a strong theoretical basis to assume that parental support affects adolescents’ depressive symptoms. For example, the experience of diminished parental support may induce feelings of emotional insecurity, which may, in turn, increase depressive symptoms [33]. A transactional perspective is critical to understanding how the adolescent not only reacts to the changes in perceived parental support but may also trigger subsequent changes in their (perception of) parents’ supportive behavior [34]. Decreases in perceived parental support may lead to increases in adolescent depressive symptoms, and increases in adolescent depressive symptoms may evoke adaptive parental support responses in the short term. Association between parental support and adolescent depressive symptoms [35]. Although parental support has a positive impact on adolescent depressed mood, it fluctuates on the time scale [36].

Parental support for physical activity is also a part of parental support. We found a significant negative correlation between parental support for physical exercise and depression. This indicates that the higher the level of encouragement, companionship, and support for exercise from parents, the lower the level of depression in their children. In other studies, social support has been repeatedly found to be negatively associated with depressive symptoms [37]. Parental support, as part of social support, may play a role distinct from other support systems, such as friend support [38]. According to the main effect model and buffering model of social support, social support can have positive effects on individuals in the face of unexpected events, regardless of whether these events are stressful. Encouragement from parents for their children’s exercise can also be understood as a form of attention, which may help reduce negative emotions in children. Parental support for their children’s exercise led to an increase in the amount of time their children spent participating in exercise, and physical activity was significantly and negatively associated with depressed mood [39]. Exercises were likely to alleviate depressive symptoms through regulation of HPA axis activity, enhancement of neurogenesis, reduction of pro-inflammatory cytokines and improvement of cardiorespiratory fitness [40]. Therefore, physical activity plays a mediating role in the effect of parental support for physical activity on adolescent depressed mood. The present study also confirms this view.

### Mediating role of physical exercise and physical self-esteem

The indirect effect of "parental support for physical exercise—physical exercise—depression" explained 49% of the total effect, making it the primary contributor to this association, highlighting the importance of physical exercise.

Similar studies have shown that children’s health behaviors are positively associated with parental support for those behaviors [41]. Reviews have also indicated that the broad support provided by parents, such as informational support (providing information about competitions and training), instrumental support (logistics and financial assistance), and emotional support (showing understanding and unconditional love), plays a crucial role in the development of young athletes and is associated with increased enjoyment, confidence, and abilities in adolescent athletes [42].

This study further emphasizes that such positive relationships exist among college students, not just children or adolescents, and play a role in promoting mental health among college students. Additionally, college students’ physical exercises are often associated with leisure, which is often used for emotional regulation. Therefore, physical exercises may be particularly helpful for college freshmen in maintaining a normal emotional state and avoiding the onset of mental illness. Furthermore, these physical exercises also help them understand the physical and interpersonal environment of college, with potential value for adaptation.

In contrast, the indirect effect of "parental support for physical exercise—physical self-esteem—depression" explained only 4% of the total effect. This is mainly due to the weak direct correlation observed between parental support for physical exercise and physical self-esteem. This result is not surprising, as the association between parental exercise support and physical self-esteem is primarily mediated by exercise, as indicated by our final model. This explains why the "parental exercise support—exercise—physical self-esteem—depression" path is longer but explains a higher proportion of the overall association (15%). This finding indicates that the role of physical self-esteem in the association between parental support for physical exercise and depression is mainly achieved through chain mediation. In this chain mediation pathway, we observed a high correlation between exercise and physical self-esteem. Similar findings have been reported in other studies, such as hockey players who engage in training having better psychological conditions and more positive self-perceptions of their bodies than non-exercising students [43]. There is also evidence that physical activity is directly or indirectly related to self-esteem [44]. Our study partly confirms these findings and further emphasizes their derivative effects on mental health.

## Limitations and future directions

This study has some limitations. Firstly, a limited sample of students was used, thus representing a restricted population. Future research should consider a more appropriate sampling approach to enhance external effectiveness. Additionally, we only used questionnaires for exercise, so the survey results could be subject to subjective reporting bias. Future research can employ devices such as accelerometers as alternatives. Furthermore, the exercise scale we used is mainly used in the Chinese population, so the results may not be easily replicated in populations of other languages. Future research may employ different measures for triangulation. Finally, we used cross-sectional data, and the regression direction between variables was determined only by assumptions. However, some variables, such as exercise and physical self-esteem, may also have reverse causal assumptions. Therefore, it is necessary in the future to explore the relationship between parental exercise support and mental health based on other theories and research designs, such as longitudinal mediation models.

## Recommendations

Based on our findings, we propose the following recommendations.

Firstly, schools and governments should advocate for parents to provide more support and encouragement for their children’s exercise activities, including encouraging them to participate in sports, exercise classes, or exercise at home. Secondly, guidance should be provided to parents on how to actively participate in their children’s physical exercises, such as exercising with them or creating a family exercise plan. Finally, parents can be provided with cognitive education about mental illness, enabling them to understand the importance of exercise and physical self-esteem in preventing depression and better supporting their children’s mental health. Lastly, schools can provide as many resources as possible for freshmen to conduct exercise, encouraging them to walk out of their dormitories and adapt to campus life more effectively.

## Conclusions

This study explored the association between parental support for physical exercise and student depression among college freshmen, as well as the chain mediation by exercise and physical self-esteem. Our findings confirmed the association between parental exercise support and student depression, with exercise being the most important mediator in this association. Additionally, the chain mediation by exercise and physical self-esteem explained a considerable amount of the overall association. These findings underscore the necessity of enhancing health and exercise education among parents.

## Supporting information

S1 File The dataset supporting the analysis. (XLSX)
